# What Outcomes Matter in Recovery in Depression?: Cross-sectional Sample of Individuals with Major Depressive Disorder in a Diverse, Safety-net Setting

**DOI:** 10.1192/j.eurpsy.2026.10601

**Published:** 2026-07-31

**Authors:** J. Lam, A. Dobbins, N. Mulvaney-Day, A. Progovac

**Affiliations:** 1 Psychiatry, Cambridge Health Alliance, Cambridge; 2 Institute of Excellence in Health Equity and Department of Population Health, New York University, New York, United States

## Abstract

**Introduction:**

Behavioral health outcome measures often emphasize symptoms, while overlooking other important outcomes, such as recovery and feeling positive. Little is known about what outcomes patients with major depressive disorder (MDD) prioritize and few studies have characterized “positive” outcome measures, such as personal recovery and flourishing.

**Objectives:**

In a sample of patients treated for MDD in an outpatient psychiatry clinic, we aim to 1) describe personal recovery outcomes most important to patients and 2) describe the relationship between measures of personal recovery, and measures of flourishing, and psychiatric symptoms and functioning.

**Methods:**

The sample included 43 patients aged 18-35 with a diagnosis of MDD receiving outpatient treatment in an urban safety net psychiatry department in Massachusetts. Participants completed an electronic survey rating outcomes most important in their recovery and to complete standardized measures of flourishing, personal recovery, functioning, and symptoms of depression and anxiety.

**Results:**

Mean age of the sample was 27.7 (*SD* = 5.2), most participants were female (*n* = 23; 53.3%), identified as non-white (*n* = 25; 59.4%), and were single (*n* = 28; 65.1%). A majority of participants had high symptoms of depression (61.9%; PHQ-8 ≥ 10), a minority had high symptoms of anxiety (37.5%; GAD-7 ≥ 10), and a minority were classified as flourishing (16.7%; Flourishing Scale ≥ 48). The top three outcomes participants ranked as “most important” in recovery were coping well with stressful events (39.5%), functioning well (34.9%), and having hopes and dreams for the future (30.2%). Flourishing and recovery questionnaires were all strongly positively correlated with one another (correlation coefficients each ≥ 0.73), and negatively correlated with symptom scales. Notably, among individuals with low depressive symptoms, 60.0% had low levels of flourishing and 31.3% had low levels of personal recovery.

**Image 1:**
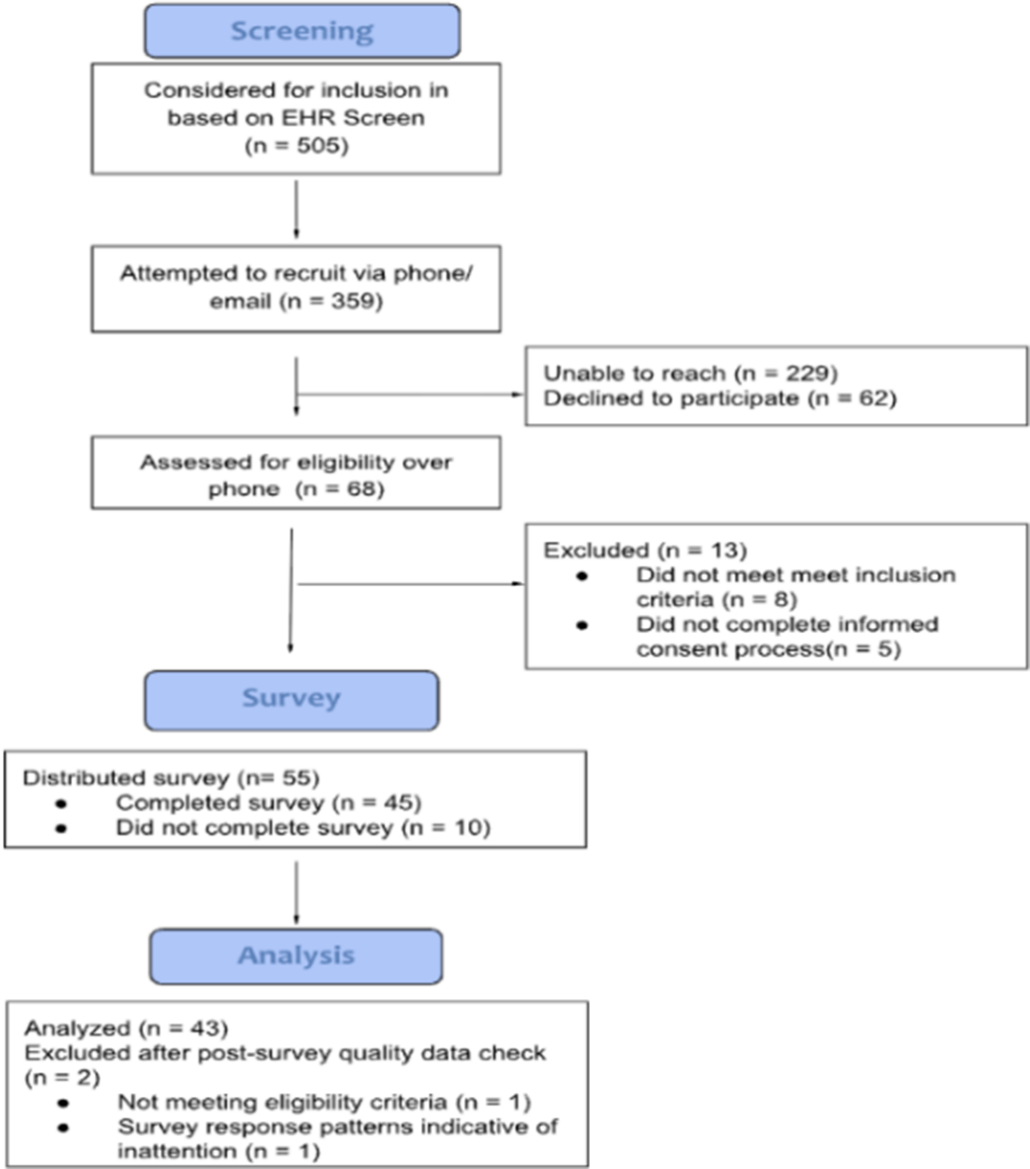
Image 1: Long description.

**Image 2:**
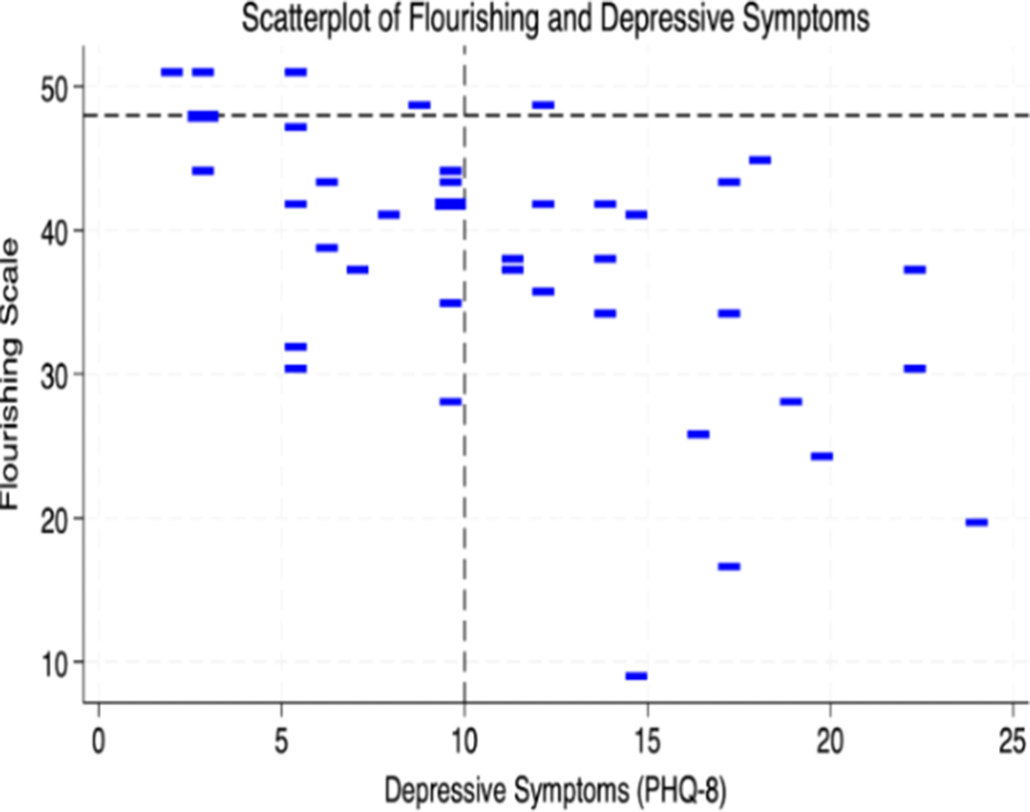
Image 2: Long description.

**Image 3:**
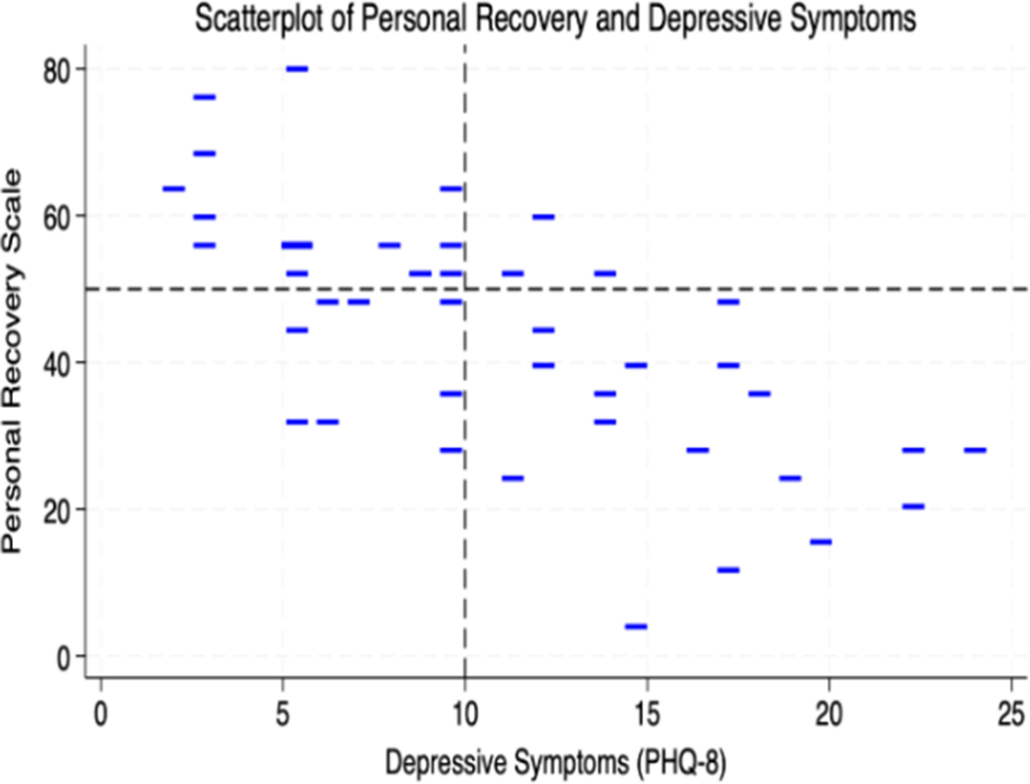
Image 3: Long description.

**Conclusions:**

Individuals with MDD value a diverse range of outcomes. These findings may suggest the need to personalize treatment toward patient-defined goals, which include more positive outcomes. Incorporating measures of flourishing and personal recovery alongside measurement of symptoms may yield a more comprehensive understanding of overall mental health.

**Disclosure of Interest:**

None Declared

## Long descriptions

### Image 1: Long description

The flowchart is divided into three main blue-shaded phases: Screening, Survey, and Analysis.

1. Screening Phase:

- Starts with ‘Considered for inclusion in based on E H R Screen (n = 505)'.

- Leads to ‘Attempted to recruit via phone/email (n = 359)'.

- A horizontal branch indicates 229 were ‘Unable to reach’ and 62 ‘Declined to participate’.

- The main path continues to ‘Assessed for eligibility over phone (n = 68)'.

- A horizontal branch shows ‘Excluded (n = 13)', consisting of 8 who ‘Did not meet meet inclusion criteria’ and 5 who ‘Did not complete informed consent process’.

2. Survey Phase:

- Follows the screening phase, starting with ‘Distributed survey (n = 55)'.

- This box notes 45 ‘Completed survey’ and 10 ‘Did not complete survey’.

3. Analysis Phase:

- The final stage is ‘Analyzed (n = 43)'.

- It notes ‘Excluded after post-survey quality data check (n = 2)', which includes 1 ‘Not meeting eligibility criteria’ and 1 with ‘Survey response patterns indicative of inattention’.

Navigate back to Image 1:.

### Image 2: Long description

The x-axis represents Depressive Symptoms P H Q 8 with a scale from 0 to 25. The y-axis represents the Flourishing Scale with a scale from 0 to 50. A vertical dashed line is positioned at x equals 10, and a horizontal dashed line is positioned at y equals 48.

Data points are represented by small blue horizontal bars. The overall trend shows a moderate negative correlation where higher depressive symptom scores generally correspond to lower flourishing scores.

* In the top-left quadrant (low depressive symptoms, high flourishing), there is a high density of data points clustered between x equals 2 and 10, and y equals 30 and 50.

* In the top-right quadrant (high depressive symptoms, high flourishing), there are very few points, with only one point appearing above the y equals 48 threshold near x equals 12.

* In the bottom-left quadrant (low depressive symptoms, low flourishing), there are no data points below y equals 28.

* In the bottom-right quadrant (high depressive symptoms, low flourishing), the data points are more widely dispersed, ranging from y equals 9 to y equals 45, showing a significant decline in flourishing as depressive symptoms increase beyond 15.

Navigate back to Image 2:.

### Image 3: Long description

The x-axis represents Depressive Symptoms P H Q 8 with a scale from 0 to 25. The y-axis represents the Personal Recovery Scale with a scale from 0 to 80. A vertical dashed line is positioned at x equals 10 and a horizontal dashed line is positioned at y equals 50, dividing the plot into four quadrants.

* The data points, represented by short blue horizontal bars, show a downward trend from the top-left to the bottom-right.

* Top-Left Quadrant (Low symptoms, High recovery): Contains a high density of points, including the highest recovery scores reaching 80 at x equals 5.

* Bottom-Left Quadrant (Low symptoms, Low recovery): Contains several points ranging from recovery scores of 30 to 50.

* Top-Right Quadrant (High symptoms, High recovery): Contains fewer points, with recovery scores mostly clustered near the 50 to 60 range.

* Bottom-Right Quadrant (High symptoms, Low recovery): Contains a wide distribution of points, with recovery scores decreasing as symptoms increase, reaching as low as 5 at x equals 15 and approximately 25 at x equals 24.

Navigate back to Image 3:.

